# Supplementary material for: The influence of survival conditions on farmer willingness to participate in non-grain conversions of cultivated land based on the SOR model
Source: PLoS One. 2025 Feb 18;20(2):e0318441. doi: 10.1371/journal.pone.0318441 (PMC11835328; doi:10.1371/journal.pone.0318441)
Supplement: Attachment — Submitted filename: Response to Reviewers.docx [file pone.0318441.s005.docx]

Dear editor,

Thank you very much for allowing us to revise our manuscript. We appreciate the editor and reviewers very much for their constructive comments and suggestions on our manuscript entitled “The influence of survival conditions on farmer willingness to participate in non-grain conversions of cultivated land based on the SOR model” (ID: PONE-D-24-22619). We carefully revised our manuscript according to the reviewers’ detailed suggestions. We hope that the revision is acceptable, and I look forward to hearing from you soon.

With best wishes,

Yours sincerely,

Hui Fan

September 14^th^, 2024

**Journal Requirements:**

1. Please ensure that your manuscript meets PLOS ONE's style requirements, including those for file naming.

**Reply**: Thank you for your nice advice. We have checked the style requirements of PLOS ONE and made some modifications. We have made corresponding modifications to the title and figure and table name in the article, and the modified content is displayed in red font in the revised manuscript.

2. We note that the grant information you provided in the ‘Funding Information’ and ‘Financial Disclosure’ sections do not match. When you resubmit, please ensure that you provide the correct grant numbers for the awards you received for your study in the ‘Funding Information’ section.

**Reply:** Thank you for your nice suggestion. The funding information will be filled in correctly when we submit the revised draft.

3. Thank you for stating the following financial disclosure:[the National Natural Science Foundation of China (Grant number 71904150, 41771438) and General Project of Humanities and Social Sciences Research in Higher Education Institutions in Henan Province（Grant number 2024-ZZJH-039].Please state what role the funders took in the study. If the funders had no role, please state: "The funders had no role in study design, data collection and analysis, decision to publish, or preparation of the manuscript." If this statement is not correct you must amend it as needed. Please include this amended Role of Funder statement in your cover letter; we will change the online submission form on your behalf.

**Reply:** According to the actual situation, we only keep one fund. It is General Project of Humanities and Social Sciences Research in Higher Education Institutions in Henan Province (Grant number 2024-ZZJH-039).

4. Please include captions for your Supporting Information files at the end of your manuscript, and update any in-text citations to match accordingly.

**Reply:** Thanks for your nice suggestions, we have updated the support information section at the end of the article.

5、Supplementary statement

The original fifth author Xiaoke Shao was changed to the third author, and the other authors were postponed. This revision has been approved by the other authors. In the revision of this paper, Xiaoke Shao participated in the writing of the article content, the drawing of the technical roadmap, the editing of the article format and the writing of the expert reply letter, and made a large contribution to the work, so the order of authors is hereby adjusted.

**Reviewer #1**

1. The abstract does not provide a concise and clear overview of the results.

**Reply:** Thanks for your suggestion. We have revised some contents of the abstract, especially the conclusion part, to improve the generality of the research conclusions. (Page 2, Lines 29-36)

2. In line 60, the use of "we" is not appropriate for academic writing. Additionally, the overall academic writing style of this manuscript requires improvement.

**Reply:** Thanks for your careful checks. We have revised several similar problems in the paper. (Page 3, Lines 58-59; Page 23 Line 481; Page 23 Lines 484-485)

3. In the second paragraph of the introduction section, the author provides a literature review of the non-grain crop (NGC) of cultivated land from four aspects. However, the influencing factors and driving mechanisms are presented in an overlapping manner, causing confusion for the reader. It is important to note that a literature review is not merely a list of sources but rather a critical evaluation and synthesis of previous works. Therefore, the author should clarify and restructure the presentation of these factors and mechanisms to enhance the logical flow and readability of the introduction.

**Reply:** Thank you for your nice suggestion. We have combined and adjusted the two parts of influencing factors and driving mechanism appropriately. (Page 3, Lines 66-75)

4. What is the scientific question of your research that is not mentioned in the introduction?

**Reply:** Thanks for your suggestion. In writing, we neglected this problem. Now, we have added scientific questions in the preface, which improves the readability of the article. (Page 5, Lines 103-105)

5. The innovation of the article is not clear enough from your literature review.

**Reply:** We sincerely appreciate the valuable comments. We have made several changes in the introduction to optimize the content and highlight the innovation of the article. (Page 5, Lines 97-111)

6. Theoretical analysis and research hypothesis section is not analyzed enough and needs to be further strengthened, the theoretical analysis needs to be supported by certain reviews and theories. It is also not recommended to put “Overview of the SOR model” in the theoretical analysis part.

**Reply:** Thank you for the suggestion. We have revised this part. The introduction of SOR model is integrated into the specific content of the paper. (Pages 6-7, Lines 123-160)

7. In Section 3.1 on Data Sources, the authors have not provided detailed validation or justification for their conclusion that the data credibility is high.

**Reply:** Thank you for your nice suggestion. It is supplemented in section 3.1.In order to ensure the accuracy of the questionnaire data, the research group adopted a one-to-one survey when conducting the questionnaire survey, and excluded the questionnaires with missing observational values, inappropriate options and other potential problems. (Page 10, Lines 227-230)

8. Nowadays, rural households are no longer choosing between the traditional agricultural or non-agricultural livelihoods, but more and more are adopting a combination of agricultural and non-agricultural livelihood strategies. On what basis the author divides these two categories of rural households, the article does not explain in detail, and the division into agricultural and non-agricultural only seems to have some subjective bias.

**Reply:** Thanks for your suggestion. In the previous interviews and investigations, the research group found that family livelihood is the main symbol to distinguish farmers in the research area, and farmers with different family livelihoods have great differences in their willingness to convert cultivated land to non-grain land. Therefore, this study explores the non-grain willingness of cultivated land from the perspective of livelihood, combined with the actual situation of the research area. (Page 5, Lines 105-110)

9. Lines 243-256, from Table 2, how the authors come up with these results, it seems that the results from the table are contrary and that there is an error in the authors' expression.

**Reply:** Thank you for your nice suggestion. The research group rechecked this part of the manuscript, and there was no problem. The reviewer's question is mainly because our expression may not be appropriate. In understanding this part of the text, Please combine Table 2. Measurement variables and descriptive statistics, especially according to the Observation Variables meaning and value of question items.

For example, the mean value (2.52) of ES1 for AL is small than that (2.72) of ES1 for NL. This shows that ES1 has a greater impact on. The mean value (2.52) of ES1 for al is small than that (2.72) of ES1 for NL. This shows that ES1 has a greater impact on AL farmers. We have revised the statistical analysis results of the perceived benefits and perceived risks of non-grain cultivation.

10. The results section lacks concise and clear presentation, and it is recommended that more precise language be used to describe the findings of the study.

**Reply:** Thanks for your suggestion. We have modified the result, deleted some unnecessary expressions, and improved the simplicity of the language expression in this part. (Page 24, Lines 491-496)

11. The discussion section, which requires the author to analyze and compare the main results in depth, is totally missing and discussion is not a reporting of results.

**Reply:** Thank you for the suggestion. We have revised this part. The previous summative content has been deleted to highlight the comparison between the research results of this paper and related results. (Page 23, Lines 481, 484-485)

**Reviewer #2:**

1. There is a lack of demonstration on the necessity of the influence of individual farmers and family factors on the non-grain conversions of cultivated land. It is suggested to increase in the introduction to reflect the significance of the research.

**Reply:** Thank you for your nice suggestion. In the introduction, we added the literature in the field of the influence of individual factors and family factors of farmer willingness to convert cultivated land to non-grain land. (Page 5, Lines 97-103)

2. It is suggested to supplement the research status of non-grain conversions of cultivated land in Henan Province.

**Reply:** Thanks for your suggestion. In the introduction, we added the literature on the current situation of non-grain conversion of cultivated land in Henan Province. (Page 5, Lines 113-115)

3. There is a lack of a full description of the abbreviations of various key words in the article.

**Reply:** Thank you for the suggestion. We have added various abbreviations to the attachments of the article. (Page 26, Lines 545-551)

4. There is a problem with the format of Hypothesis 7 last sentence, please correct it. In addition to this, there are other places in the article also exist the same problem, please check carefully.

**Reply:** Thank you for your nice suggestion. We corrected this mistake, and also corrected other similar mistakes in the paper. (Page 9, Lines 205-206)

5. The technical method part of the article is not clear enough, and it is recommended to add a technical flow chart as a whole.

**Reply:** Thanks for your suggestion. We have added a technical flow chart to make the technical methods section clearer. (Page 9, Line 210, Figure 2)

6. Whether the SOR method is appropriate and reasonable here, it is recommended to supplement the discussion.

**Reply:** Thank you for the suggestion. We explain the application of SOR in this article. (Pages 6-7, Lines 136-141)

7. It is necessary to emphasize the correspondence between policy impact and key conclusions.

**Reply:** Thank you for your nice suggestion. We have revised the policy recommendations section to provide a better correspondence between policy impact and conclusions. (Page 25, Lines 512-519, 527-533)
