# Supplementary material for: The influence of survival conditions on farmer willingness to participate in non-grain conversions of cultivated land based on the SOR model
Source: PLoS One. 2025 Feb 18;20(2):e0318441. doi: 10.1371/journal.pone.0318441 (PMC11835328; doi:10.1371/journal.pone.0318441)
Supplement: Attachment — Submitted filename: Response_to_Reviewers_auresp_2.docx [file pone.0318441.s006.docx]

Dear editor,

Thank you very much for allowing us to revise our manuscript. We appreciate the editor and reviewers very much for their constructive comments and suggestions on our manuscript entitled “The influence of survival conditions on farmer willingness to participate in non-grain conversions of cultivated land based on the SOR model” (ID: PONE-D-24-22619R1). We carefully revised our manuscript according to the reviewers’ detailed suggestions. We hope that the revision is acceptable, and I look forward to hearing from you soon.

With best wishes,

Yours sincerely,

Hui Fan

6^th^ January, 2025

Journal Requirements:

Please review your reference list to ensure that it is complete and correct. If you have cited papers that have been retracted, please include the rationale for doing so in the manuscript text, or remove these references and replace them with relevant current references.

Thanks for your suggestion. We have checked the literature according to your request and it all meets the requirements, no need to change it.

In response to the reviewers' comments, we have provided the following answers to the relevant questions that require a response

2. Is the manuscript technically sound, and do the data support the conclusions?

Drawing on relevant articles, we observe the corresponding principles in the process of questionnaire design, questionnaire survey, and model construction in order to ensure that the scientific validity of the findings is guaranteed

3. Has the statistical analysis been performed appropriately and rigorously?

In order to make the statistical analysis more scientific and rigorous, we used a questionnaire survey method combining stratified sampling and random sampling. The process of statistical analysis of the data also strictly adhered to the requirements of statistics.

5. Is the manuscript presented in an intelligible fashion and written in standard English?

We have invited scholars with experience of studying in the United States to revise the language of the article during the writing process, and in addition we have asked a specialized AJE agency to polish the language of this article.

6. Reviewer #3: The authors have improved their paper adding important parts. Still I have doubts concerning the impact of their research. The case study, even based on 650 questionnaires, could be not essential for the China Policy because involves only one Province. Also for the world this could be a very specific case. Authors tried to explain in their specific way a simple case that could not be useful. Maybe this must be added at the Conclusion chapter. Or specified since the beginning. The perceived benefit is always first, that is why the research must be orientated in other direction.

Thank you for your suggestions. We understand your concerns about the generalizability and practical impact of the study. Indeed, our case study is limited to a single province, so its implications for policy in China may be limited, and there are some limitations for global replication. Through a detailed examination of Henan Province, a major food-producing region, this study is dedicated to exploring the factors influencing the willingness to NGC of cultivated land and its specificities, with a view to providing theoretical support and lessons for the governance of defragmentation of arable land in other similar regions. We have already pointed this out in the introduction (lines 122-126) and in the conclusion of this paper (lines 501-503). And we state the scope of application of the conclusions of this study, which mainly applies to China's main grain-producing provinces.
